# Cost effective improvement in the protocol for detection of haemoglobin variants –a step forward in quality assurance

**DOI:** 10.1186/1756-0500-6-192

**Published:** 2013-05-09

**Authors:** Natasha Ali, Mohammad Khurshid

**Affiliations:** 1 Department of Pathology and Microbiology/Oncology, The Aga Khan University and Hospital, Stadium Road, P.O Box 3500, Karachi, 74800, Pakistan

**Keywords:** HPLC, Peripheral blood film, Haemoglobinopathy

## Abstract

**Background:**

We report the results of a cost effective improvement in the protocol for detection of haemoglobin variants which incorporates the findings of peripheral blood film along with the results of HPLC.

**Findings:**

A total of n = 10,844 samples were received from January 2011 till August 2011. Diagnosis of haemoglobinopathy was made in n = 1123 samples while other abnormalities included iron deficiency anaemia, megaloblastic anaemia, malarial parasite, autoimmune haemolytic anaemia and G6PD deficiency (n = 2473).

**Conclusion:**

We diagnosed 23% of abnormalities other than haemoglobinopathy by reviewing peripheral smear of samples received for detection of haemoglobin variants. This resulted in providing proper diagnosis to the referring physician without increment in cost.

## Findings

We report the results of a cost effective improvement in the protocol for haemoglobin variant detection which incorporates the findings of peripheral blood film along with haemoglobin and its variants. Modern healthcare these days requires multidisciplinary approaches to patient centred management. Similarly, the diagnosis of haemoglobin disorders requires combination of techniques [1]. Haemoglobin electrophoresis is still the most common technique for initial detection and characterisation of abnormal haemoglobin variants [2]. However, high performance liquid chromatography is increasingly taking its place. At our laboratory, samples sent for detection of abnormal haemoglobin variants are performed on high performance liquid chromatography (HPLC). In the year 2001, the College of American Pathologists committee members concluded that physician’s review of peripheral smear comprised an essential component of proper patient care. As part of implementing continuous improvement in quality assurance, we review peripheral blood films of all samples that are received for HPLC. These blood films are reviewed irrespective of physician’s request, in return providing extra results to help in diagnosis with no increase in cost.

**Figure 1:**
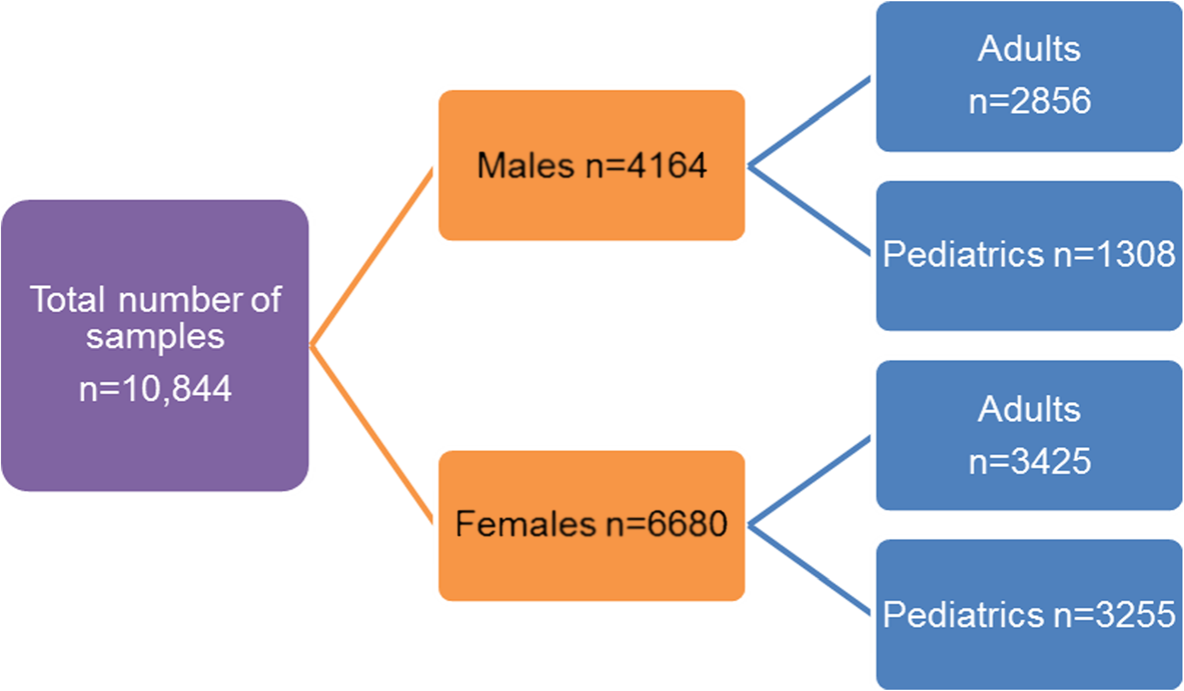
Distribution of adult and paediatric patients in samples received for haemoglobin electrophoresis.

This study was given exemption from ethical approval by the institution’s ethical review committee (2416-Pat-ERC-12). From January 2011 till August 2011, we reviewed peripheral blood films of all samples received for HPLC to determine the frequency of diagnosing abnormalities other than haemoglobinopathy. Venous blood (5 ml) was collected in EDTA and HPLC was performed on Bio Rad variant for all samples. Total number of samples received during the study period were n = 10,844 out of which n = 4164 were males and n = 6680 were females. Further breakdown of adult and paediatric patients is given in Figure 1. Out of n = 10,844, n = 1123 were diagnosed as haemoglobinopathy and n = 7248 were normal. The remaining i.e. n = 2473 had a diagnosis other than haemoglobinopathy.

Iron deficiency anaemia was the most common pathology noted (n = 2238) followed by malarial parasite (n = 104). Other abnormalities (n = 235) included acute leukemia, G6PD deficiency, megaloblastic anaemia, malarial parasite and autoimmune haemolytic anaemia (Table 1). The appropriate investigations for further confirmation of the peripheral blood smear findings were suggested in the report. These included serum ferritin levels for iron deficiency anemia and enzyme assays for G6PD deficiency. For autoimmune haemolytic anemia, direct antihuman globulin test was performed free of charge and results were incorporated in the final report.

**Table 1:** Breakdown of other results n = 2473

**Abnormality**	**n**
**Malarial Parasite**	104
**Acute Leukemia**	28
**G6PD Deficiency**	11
**Megaloblastic Anaemia**	83
**Autoimmune Haemolytic Anaemia**	9
**Iron Deficiency Anaemia**	2238
**Total**	**2473**

By following this protocol, we diagnosed 23% of abnormalities which were other than haemoglobinopathy. This resulted in providing the right diagnosis to the requesting physician hence better service without cost increment. The next step would be to encourage adaptation of this protocol at the national level which would result in timely diagnosis, improved quality of laboratory data and better patient management.

## Competing interests

The authors declare that they have no competing interests.

## Authors’ contributions

NA drafted the manuscript, collected data. MK critically analyzed the manuscript. Both authors read and approved the final manuscript.

## References

[B1] Sangkitporn S Sangkitporn SK Tanjatham S Suwannakan B Rithapirom S Yodtup C Yowang A Duangruang S Multicenter validation of fully automated capillary electrophoresis method for diagnosis of thalassemias and hemoglobinopathies in Thailand Southeast Asian J Trop Med Public Health 2011 42 5 1224 1232 22299449

[B2] Fucharoen S Winichagoon P Wisedpanichkij R Sae-Ngow B Sriphanich R Oncoung W Muangsapaya W Chowthaworn J Kanokpongsakdi S Bunyaratvej A Piankijagum A Dewaele C Prenatal and postnatal diagnoses of thalassemias and hemoglobinopathies by HPLC Clin Chem 1998 44 4 740 748 9554484

